# Vascular cognitive impairment and dementia: Mechanisms, treatment, and future directions

**DOI:** 10.1177/17474930241279888

**Published:** 2024-09-16

**Authors:** Vincent Chung Tong Mok, Yuan Cai, Hugh S Markus

**Affiliations:** 1 Lau Tat-chuen Research Centre of Brain Degenerative Diseases in Chinese, Therese Pei Fong Chow Research Centre for Prevention of Dementia, Lui Che Woo Institute of Innovative Medicine, Gerald Choa Neuroscience Institute, Li Ka Shing Institute of Health Science, Division of Neurology, Department of Medicine and Therapeutics, Faculty of Medicine, The Chinese University of Hong Kong, Prince of Wales Hospital, Hong Kong SAR, China; 2 Stroke Research Group, Department of Clinical Neurosciences, University of Cambridge, Cambridge, UK

**Keywords:** Vascular cognitive impairment, small-vessel disease, Alzheimer’s disease, stroke, dementia, vascular risk factors

## Abstract

Worldwide, around 50 million people live with dementia, and this number is projected to triple by 2050. It has been estimated that 20% of all dementia cases have a predominant cerebrovascular pathology, while perhaps another 20% of vascular diseases contribute to a mixed dementia picture. Therefore, the vascular contribution to dementia affects 20 million people currently and will increase markedly in the next few decades, particularly in lower- and middle-income countries.

In this review, we discuss the mechanisms of vascular cognitive impairment (VCI) and review management. VCI refers to the spectrum of cerebrovascular pathologies that contribute to any degree of cognitive impairment, ranging from subjective cognitive decline, to mild cognitive impairment, to dementia. While acute cognitive decline occurring soon after a stroke is the most recognized form of VCI, chronic cerebrovascular disease, in particular cerebral small-vessel disease, can cause insidious cognitive decline in the absence of stroke. Moreover, cerebrovascular disease not only commonly co-occurs with Alzheimer’s disease (AD) and increases the probability that AD pathology will result in clinical dementia, but may also contribute etiologically to the development of AD pathologies.

Despite its enormous health and economic impact, VCI has been a neglected research area, with few adequately powered trials of therapies, resulting in few proven treatments. Current management of VCI emphasizes prevention and treatment of stroke and vascular risk factors, with most evidence for intensive hypertension control. Reperfusion therapies in acute stroke may attenuate the risk of VCI. Associated behavioral symptoms such as apathy and poststroke emotionalism are common. We also highlight novel treatment strategies that will hopefully lead to new disease course-modifying therapies. Finally, we highlight the importance of including cognitive endpoints in large cardiovascular prevention trials and the need for an increased research focus and funding for this important area.

In our rapidly aging society, the management and research of cerebrovascular disease (CVD) are of global importance not only because of its association with stroke but also because of its contribution to cognitive impairment. Vascular cognitive impairment (VCI) refers to the entire spectrum of vascular brain pathologies that contribute to any degree of cognitive impairment, ranging from subjective cognitive decline, to mild cognitive impairment (MCI), to dementia.^1^ While acute cognitive decline occurring soon after a stroke is the most recognized form of VCI, CVD, in particular cerebral small-vessel disease (SVD), can also induce insidious cognitive decline in the absence of stroke.^2^ Moreover, studies conducted over the recent decades have shown that CVD not only commonly co-occurs with Alzheimer’s disease (AD) and increases the probability that AD pathology will result in clinical dementia, but may also contribute etiologically to the development of AD pathologies. Since recent forecasts predict that the number of people living with dementia will triple from around 57.4 million currently to 152.8 million by 2050 and the greatest increase will be at low-income and middle-income countries (LMICs) where control of CVD is suboptimal,^3^ tackling CVD is of paramount importance in combating this coming dementia tsunami. In this review, we provide an update on the mechanisms and management of VCI, as well as highlighting a number of articles published in this special edition of the International Journal of Stroke (IJS) on the vascular contribution to dementia.

## Mechanisms of VCI

### Poststroke cognitive impairment

Stroke commonly leads to acute onset of cognitive decline soon after the stroke event. Systematic reviews show that the prevalence of poststroke MCI and dementia are around 38% and 18.4%, respectively.^4–6^ Age and vascular risk factors/cardiovascular diseases (e.g. hypertension, hyperlipidemia, diabetes, atrial fibrillation (AF), physical inactivity, smoking, nutrition, obesity, and air pollutants) explain the majority of stroke risk. Whether the stroke event induces cognitive impairment depends on a complex interplay between the features of the stroke lesion itself and the brain resilience of the individual. Large lesions or lesions located at “strategic” sites (e.g. left frontotemporal lobe, left thalamus, or right parietal lobe) are more prone to result in cognitive impairment.^4,7,8^ Brain resilience depends on a host of factors, including pre-existing brain diseases (e.g. AD and SVD), cognitive reserve (e.g. educational level and cognitive activity), and other demographic or medical conditions (e.g. age, diabetes, and frailty).^9^ Even a small lesion located in a non-strategic region, or a transient ischemic attack, may trigger poststroke cognitive impairment in someone with low brain resilience. The importance of pre-existing brain disease is emphasized by a meta-analysis by Ball and colleagues who in this month’s issue of IJS show that that pre-existing extent of magnetic resonance imaging (MRI) markers of SVD (white matter hyperintensities (WMHs), cerebral microbleeds (CMBs), and brain atrophy) present at the acute stage of stroke were associated with poststroke cognitive impairment.^10^ It is notable that in contrast they did not find an association with features of the acute stroke lesion itself. In another study, also published in this issue of IJS, using the data from the Meta VCI Map consortium (n = 1568), Coenen et al.^11^ found that apart from the total volume of pre-existing WMH volume, WMH located at “strategic” white matter tracts (left anterior thalamic radiation, forceps major) was also associated with poststroke cognitive impairment. This finding implies that the concept of “strategic” lesions extends beyond acute stroke lesions and involves pre-existing WMH as well. It emphasizes the importance of white matter damage in causing cognitive impairment, a view reinforced by results from diffusion tensor imaging (DTI) showing that diffuse white matter damage correlates with the degree of cognitive impairment.^12^ Further studies using DTI data to construct brain networks have shown the central role of network disruption in mediating the effect of multiple different SVD pathologies, including WMH and lacunar infarcts, on causing VCI.^13,14^

**Figure 1.**
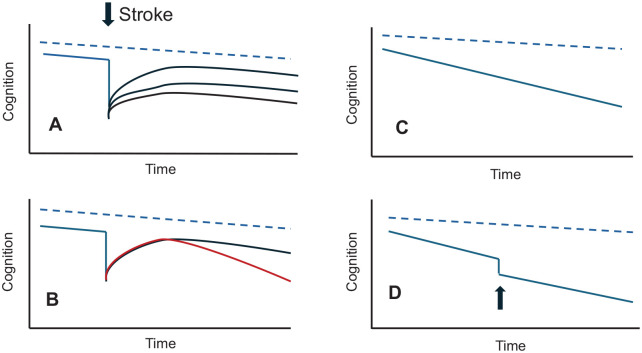
Trajectories of different types of vascular dementia. In all figures, the dotted blue line represents cognitive decline with age in the absence of cerebrovascular disease: (A) Following a stroke cognition declines, and then recovers to a variable extent. The extent of the poststroke cognitive decline will depend both on the site and extent of the stroke, as well as brain resilience. Brain resilience depends on pre-existing brain diseases (e.g. AD and SVD), cognitive reserve (e.g. educational level and cognitive activity), and other demographic or medical conditions (e.g. age, diabetes, and frailty); (B) following stroke cognition can continue to decline in the longer term at a rate faster than that prestroke as illustrated by the red line; (C) cerebrovascular disease can result in gradual decline in cognition in the absence of stroke. This pattern is particularly seen with SVD; (D) other insults such as delirium and intercurrent infection (arrowed) can result in a decline in cognition which does not return to the pre-insult level. Source: © Hugh Markus.

It is also noteworthy that even after the cognitive decline associated with an acute stroke has settled, the long-term rate of cognitive decline over subsequent years is faster when compared to that in stroke-free individuals and such a decline occurs even in the absence of recurrent stroke^25,26^ (Figure 1). One factor driving this delayed-onset insidious cognitive decline is that the stroke episode triggers the progression of pre-existing brain diseases (e.g. AD and SVD). Other possible mechanisms may include concurrent medical conditions (e.g. diabetes and frailty), or stroke triggering an immune response that leads to on-going inflammation.^9,27,28^

From a management perspective, it is important to determine the contribution of various brain diseases (e.g. vascular, AD, and mixed vascular/AD) in accounting for the cognitive impairment in an individual patient. Clinical guidelines from the American Heart Association/American Stroke Association (AHA/ASA)^1^ and the Diagnostic and Statistical Manual of Mental Disorders–Fifth Edition (DSM-V)^29^ have been proposed to determine whether the cognitive impairment has a strong vascular component. Both criteria suggest that if (1) the cognitive impairment has a clear temporal relationship with the cerebrovascular event, (2) neuroimaging shows significant relevant CVD lesions, and (3) there is no apparent progressive cognitive decline before or after the cerebrovascular event which may imply underlying neurodegenerative diseases (e.g. AD), then the cognitive impairment probably has a predominant vascular etiology. Yet these criteria have not yet been validated using neuroimaging. In this issue of IJS, Folloso and colleagues performed MRI and amyloid positron emission tomography (PET) imaging in 186 subjects recruited from a memory clinic and found that both criteria can identify cases with predominant vascular pathologies with little or no concomitant amyloid pathology. Rates of amyloid positivity of “probable” vascular MCI (3.8%) and “probable” vascular dementia (15%) were identical between the two criteria and were significantly lower than that of “possible” vascular MCI and “possible” vascular dementia, respectively.^30^

Concurrent medical conditions may also affect poststroke outcome. Also in this issue, using the Japan Stroke Databank (n = 56,230), Miwa et al.^31^ found that functional outcome following ischemic or hemorrhagic stroke was significantly associated with body mass index (BMI), in that a lower BMI (<18.5 kg/m2) was associated with greater disability and mortality, while a high BMI (>30 kg/m^2^) similarly associated with a worse outcome after large artery atherosclerosis disease (LAD)-related stroke. Although this study did not specifically look at cognitive outcome, it is possible that poststroke cognitive outcome could be worse among those who are underweight. A previous systematic review suggested that although mid-life obesity is associated with late-life dementia, being underweight was found to be associated with dementia when the BMI was assessed a few years before dementia diagnosis.^32^ Low body weight likely reflects a poor underlying physical condition, which could be related to underlying brain diseases (e.g. AD and SVD) at the predementia phase, thereby increasing the risk of poorer functional outcome poststroke. To date, studies on the association between body weight and poststroke cognitive impairment showed conflicting results.^33,34^ Further study is needed to investigate the association between BMI and poststroke cognitive impairment.

Given that poststroke cognitive impairment depends on multiple factors, researchers have attempted to design a clinical model that can predict its risk.^35,36^ In this issue of IJS, Ashburner and colleagues present a risk score based on simple clinical and demographic information that are easily available on electronic health records that can identify patients at risk of poststroke cognitive impairment more than 5 years.^37^ The C-statistic for predicting poststroke cognitive impairment was 0.731 in the internal validation cohort (n = 1925) and 0.724 in the external validation cohort (n = 2237). This risk score includes the following data: age, type of insurance, mobility problems, prior falls, delirium, peripheral vascular disease, Parkinson’s disease, depression, chronic kidney disease, abnormal weight loss and anorexia, and discharge to facility. Such a risk score could be used to risk stratify and identify individuals at increased risk for poststroke cognitive impairment for preventive efforts.

### SVD—the predominant pathology underlying VCI

**Figure 2.**
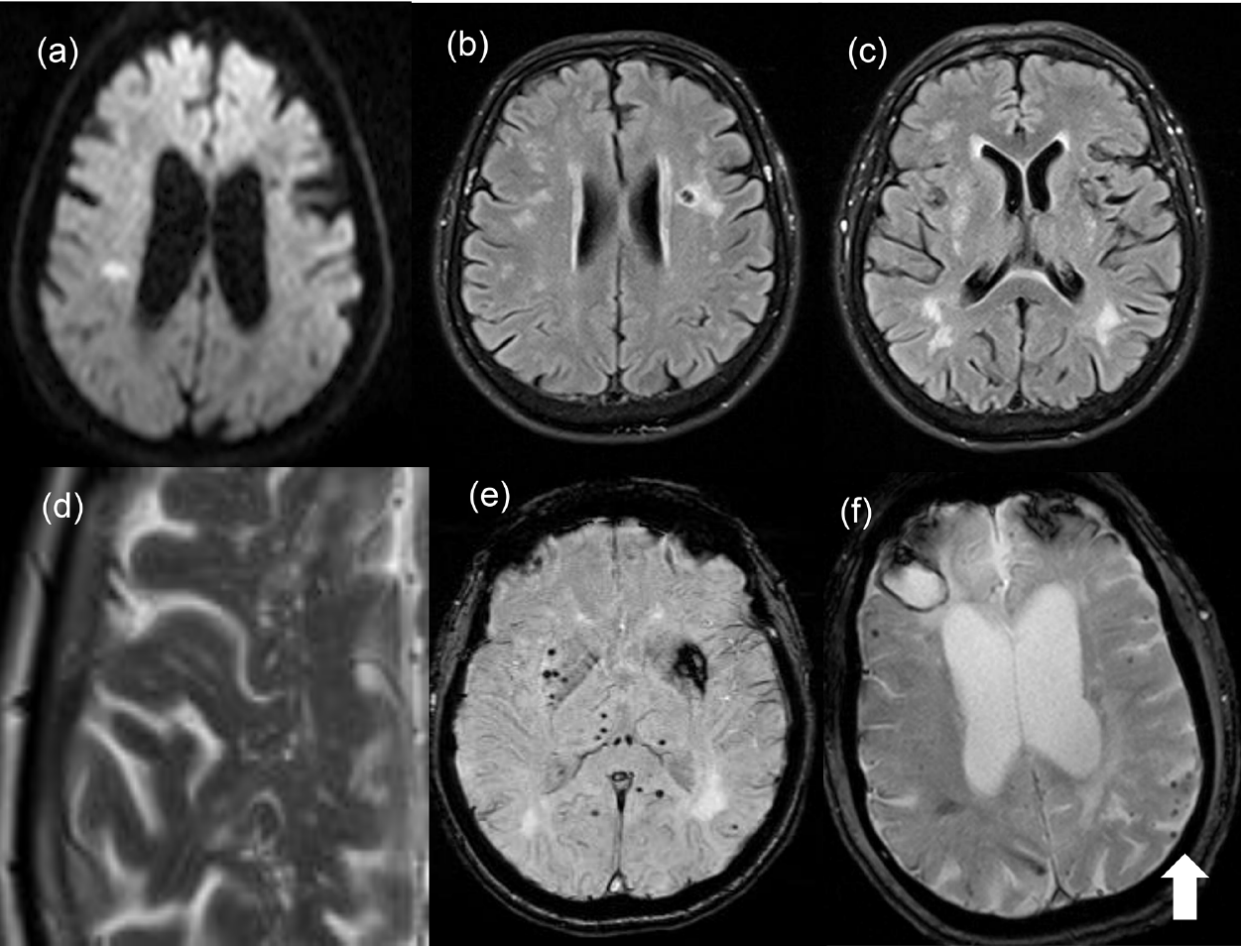
MRI appearances of SVD: Note the right side of the brain is represented on the left-hand side of the images: (A) An right hemisphere acute lacunar infarct visible as high signal on diffusion-weighted imaging; (B) a chronic lacunar infarct on the left visible as an area of low signal on FLAIR MRI; (C) white matter hyperintensities particularly in the posterior periventricular white matter; (D) a close-up of a T2-weighted MRI showing enlarged PVSs; (E) gradient echo MRI from a patient with sporadic hypertensive SVD showing multiple CMB visible as small dots of low intensity. These as predominantly subcortical, in contrast to mainly cortical CMB seen in CAA. There is also a larger area of low density in the left hemisphere representing changes from an old subcortical intracerebral hemorrhage; (F) gradient echo MRI from a patient with CAA showing multiple cortical CMB (arrowed), as well as a right frontal lobar intracerebral hemorrhage. © Hugh Markus.

Sporadic non-amyloid cerebral SVD (arteriosclerosis) and sporadic cerebral amyloid angiopathy (CAA) are the two commonest subtypes of SVD and both can induce cognitive impairment even in the absence of causing overt stroke episodes. SVD is considered the most common substrate for VCI. SVD affects the perforating arterioles and venules, as well as capillaries that encompass the neurovascular unit and blood–brain barrier (BBB). Aging, vascular risk factors (e.g. hypertension), increase in pulsatile flow being transmitted to the cerebral small vessels secondary to stiffness of systemic arterial vasculature,^38^ and/or genetic factors^2,39–41^ may lead to various abnormal structural and functional changes in the cerebral small vessels (e.g. luminal narrowing/occlusion, vessel rupture, endothelial dysfunction, BBB leakage, neuroinflammation, and impairment of glymphatic-meningeal lymphatic system), leading to varying severity levels of tissue damage, and secondary neurodegeneration, and eventually stroke and/or cognitive impairment. In general, cognitive impairment is associated with increasing extent or severity of tissue damage, lesions located at strategic regions or disrupting network connectivity, and secondary neurodegeneration. Traditionally on structural MRI, these SVD-related tissue damage may be visualized as recent small subcortical infarcts, WMH, lacunes, CMBs, enlarged perivascular space (PVS), and brain atrophy^42^ (Figure 2). Other research-orientated MRI methods can detect more subtle brain white matter alterations on DTI, BBB leakage using contrast-enhanced imaging, and impaired cerebrovascular reactivity.^43,44^ Sporadic SVD commonly affects small vessels supplying deep subcortical, brainstem, or cerebellar regions. Apart from stroke (lacunar syndromes, deep intracerebral hemorrhage (ICH)) and cognitive impairment, other clinical manifestations of SVD include Parkinsonism and behavioral problems including apathy.^45^

The severity of SVD-related lacunar stroke is commonly mild and is not always visible on computed tomography (CT) imaging particularly in the acute setting. It is much better seen on MRI but this is not always available making clinical tools for diagnosis useful particularity in less-resourced settings. In this issue, Arba and colleagues validated such a clinical score to identify lacunar stroke in the acute setting based on the presence of a lacunar syndrome and a low National Institute of Health Stroke Scale score (<7). Using data from the WAKE-UP trial (n = 503), the lacunar score had a very good specificity (0.82) and negative predictive value (0.84). However, the sensitivity (0.44) and positive predictive value (0.39) were low. Overall, the score could reliably exclude up to 9 of 10 patients with acute non-lacunar infarct.^46^ This simple score may not only help to guide patient selection in clinical trials in hyperacute stroke setting, but it may also help to facilitate research on SVD in low-income regions where MRI is not easily accessible.

Recently, two more MRI SVD lesion types have been more widely recognized and were added to the recent updated neuroimaging standards for SVD research guidelines,^43^ namely cortical cerebral microinfarcts (CCMs)^47^ and incidental diffusion-weighted imaging (DWI)-positive lesions.^47^ In a recent review in the IJS, Huang et al. discuss potential mechanisms by which these minute CCMs may induce cognitive impairment. The authors suggest mechanisms include (1) causing loss of function of neural tissues surrounding the microinfarct core with interruption of cortico-cortical and cortico-subcortical circuits, (2) disruption of brain structural network connectivity, and/or (3) secondary remote cortical neurodegeneration.^47^

Sporadic CAA is associated with age and is relatively less associated with vascular risk factors when compared to sporadic SVD. Genetic factors, namely apolipoprotein E (APOE) ε4/ε2, were found to be associated with CAA and may contribute to the deposition of beta-amyloid (Aβ) in cerebral small vessels. CAA affects small vessels supplying cerebral cortex and leptomeninges. Since Aβ weakens the vessel wall, CAA commonly results in recurrent lobar ICH, convex subarachnoid hemorrhage, and cortical superficial siderosis. Other MRI lesions similar to that of sporadic SVD (e.g. WMH, CMB, and enlarged PVS) are also common in CAA,^48^ and these can induce cognitive impairment even in the absence of stroke, with a recent study reporting that altered white matter diffusivity on DTI, cerebral atrophy, and altered cerebrovascular reactivity accounted for about half the effect of CAA on cognition.^49^ It has been suggested that sex may also influence the clinical presentation of CAA, in that male sex was associated with an earlier age of first ICH and a more hemorrhagic disease course than women possibly because man has high greater global Aβ load than women.^50^ In this issue, Koemans and colleagues investigated such a hypothesis using histopathological markers associated with Aβ burden and hemorrhage in two autopsy databases (n = 6139). However, they failed to find any sex difference with respect to vascular Aβ (CAA) and even observed significantly more CMB on *ex vivo* MRI in women. Yet, they found a higher cortical iron in men. They concluded that previously observed sex differences in hemorrhage onset and progression in CAA patients are likely not due to difference in global CAA severity between men and women. Other factors, such as vascular remodeling, may contribute as unique pattern of vascular remodeling is associated with cortical superficial siderosis.^51^

### The interaction of vascular risk and CVD with AD

**Figure 3.**
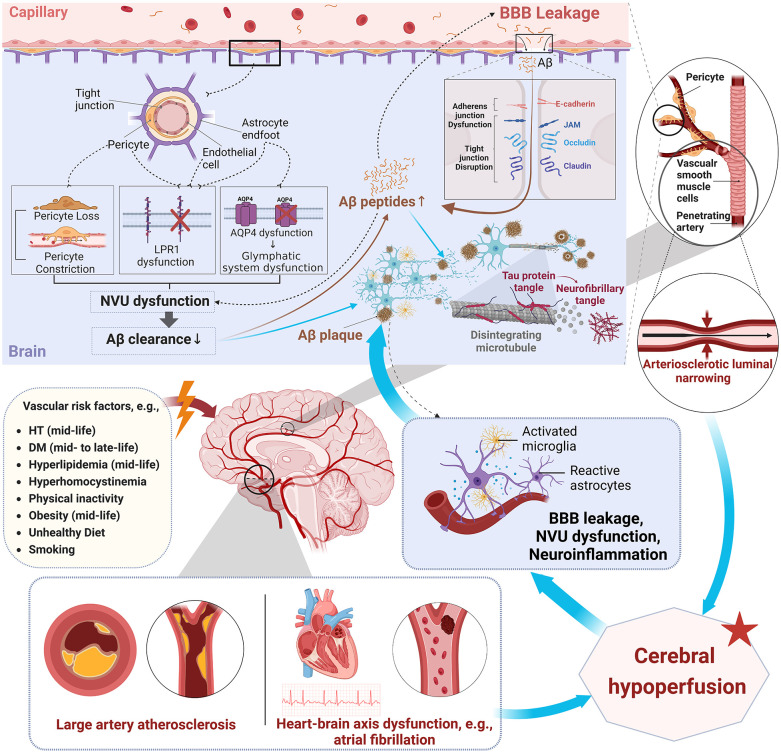
Interplay of vascular dysfunction and neurodegenerative pathways in Aβ and tau pathology. This figure highlights the complex relationship between vascular dysfunction and pathways leading to the accumulation of Aβ and tau protein formation. Modifiable vascular risk factors probably cause dysfunction of the neurovascular unit (NVU), leakage of the BBB, and neuroinflammation. The disruption of tight junctions facilitates the increased entry of Aβ from the bloodstream into the brain. Features of NVU dysfunction, such as pericyte constriction and loss, along with AQP4 mis-localization and decreased levels of LRP1, lead to inefficient Aβ clearance and promote its accumulation in the brain. In addition, activated microglia and reactive astrocytes intensify neuroinflammation, which further exacerbates BBB leakage and NVU dysfunction. All these changes contribute to the formation of Aβ plaques and the hyperphosphorylation of tau proteins, resulting in neurofibrillary tangles. The figure also connects vascular risk factors with changes in vascular smooth muscle cells that lead to arteriosclerotic luminal narrowing. Furthermore, conditions like LAD and heart–brain axis dysfunction, exemplified by atrial fibrillation, also aggravate cerebral hypoperfusion. The cerebral hypoperfusion may exacerbate NVU dysfunction, BBB leakage, and neuroinflammation, further promoting Aβ deposition and tau pathology. Aβ deposition in turn reverberates with neuroimmune and vascular dysfunction, further exacerbating the progression of this process. Aβ: amyloid-beta; BBB: blood–brain barrier; NVU: neurovascular unit; AQP4: aquaporin 4; LRP1: low-density lipoprotein receptor-related protein 1; HT: hypertension; DM: diabetes mellitus.

The latest Lancet standing Commission reported that vascular risk factors (e.g. mid-life hypertension, mid-life hyperlipidemia, and mid-life diabetes) are associated not only with all-cause dementia but also with AD.^52^ Early pathology studies showed that various CVD lesions (e.g. arteriosclerosis, lacune, white matter changes, and LAD large infarcts) commonly co-occur in the majority of AD patients (~80%) and exert additive or interactive effects in inducing cognitive impairment.^53,54^ In addition, preclinical and clinical evidence showed that SVD is an early initiating event in the development of Aβ. Longitudinal studies with serial amyloid PET imaging showed that arterial stiffness^55^ and WMH on MRI^56^ were associated with increased Aβ deposition. A number of mid-life vascular risk factors (e.g. hypertension, diabetes, hyperlipidemia, obesity, and smoking) are associated with greater amyloid deposition in late life,^57^ while greater physical activity and lower vascular risk independently attenuated the negative association of Aβ burden with cognitive decline and neurodegeneration in asymptomatic individuals.^58^ Recent longitudinal studies showed that vascular risk factors interact with Aβ to promote cognitive decline by accelerating tau accumulation^59^ and brain atrophy.^60^ The presence of retinal microvasculopathy (e.g. increased retinal arteriolar tortuosity) was found to associate with incident dementia more than 10 years.^61^ A recent pathology study showed that higher healthy lifestyle scores (physical activity, smoking status, diet quality, cognitive activity, alcohol consumption) were associated with lower Aβ load and better cognitive functioning proximate to death.^62^ It is most likely that BBB integrity, neurovascular unit function, and the glymphatic system are compromised by aging and vascular risk factors, leading to reduced clearance or increased influx of Aβ, triggering the pathological cascades of AD (Figure 3).^63–66^

An early pathology study observed an association between LAD and AD pathologies^67^ while clinical studies showed an association between AF and AD.^68^ A recent clinical study further showed an association between atrial volume/atrial cardiopathy with increased cerebral Aβ load.^69^ A possible explanation for such findings is that LAD and heart–brain dysfunctions may associate with cerebral hypoperfusion, hence affecting BBB integrity and neurovascular unit function, resulting in Aβ/tau accumulation (Figure 2).^70^

## Management of VCI

Considering its global health importance, there is a paucity of well-designed adequately power clinical trials to guide therapy in poststroke cognitive impairment and vascular dementia. In this section, we review the available data and consider how treatments for acute stroke could reduce poststroke cognitive impairment, long-term preventive therapies for VCI and vascular dementia, and symptomatic therapies for those already suffering from VCI and vascular dementia, and finally management of associated behavioral symptoms such as apathy and poststroke emotionalism.

### Acute stroke treatments

#### Acute ischemic stroke and reperfusion therapy

Given stroke commonly induces acute onset cognitive impairment, any measures (e.g. intravenous thrombolytic (IVT) and endovascular thrombectomy (EVT)) that can mitigate brain damage during the acute stage should reduce poststroke cognitive impairment. The Virtual International Stroke Trial (n = 6268) suggested that cognitive outcome was possibly better in thrombolysed compared with non-thrombolysed patients with ischemic stroke.^71^ Secondary analysis of the REVASCAT (n = 206) showed that Solitaire thrombectomy was associated with better cognitive performance than the best medical treatment at 3 months and 1 year after stroke.^72^ The most recent secondary analysis of the ESCAPE trial (n = 315) showed that the odds of favorable cognitive outcome was higher with EVT than with control.^73^

In this issue of IJS, Gallucci et al.^74^ investigated the prevalence of early poststroke cognitive impairment among survivors with first-ever anterior circulation ischemic stroke without prestroke cognitive decline, the majority of whom had received acute reperfusion therapy. Although they found that the rate of cognitive impairment remained high (69.3%), they noted that when compared to previous studies, the prevalence of aphasia and neglect in this study (18%) was much lower than that of previous studies (15–63%). They found that risk factors for reduced poststroke cognitive impairment were lesser stroke severity, right hemispheric lesions, higher education, and the absence of hyperlipidemia. Moreover, poststroke cognitive impairment was associated with poor functional outcome at 3 months poststroke. In another observational study, using the Ontario Stroke Registry, the authors found that among patients with first-ever ischemic stroke who had not been previously diagnosed with dementia, thrombolysis was associated with a 24% reduced rate of dementia.^75^

A common clinical question is whether to perform reperfusion therapy in patients with pre-existing cognitive impairment and dementia. It has been suggested that they may benefit less, but also that the pathology causing dementia might increase the risk of bleeding after thrombolysis, particularly in patients with CAA. These patients were often excluded from clinical trials meaning there are less data to guide us. In this issue of IJS, Fouzi and colleagues examined this question by performing a meta-analysis of five observational studies of IVT use in patients with (n = 1078) and without prestroke dementia (n = 2805).^76^ They found no significant differences in favorable outcome, mortality, ICH, and symptomatic ICH for patients undergoing IVT with prestroke dementia versus those without. One EVT study (n = 615 with dementia vs n = 9600 without) also found no significant differences in outcomes apart from an increased odds of ICH for those with pre-existing dementia. Overall, they concluded that there are no substantial safety issues in the use of IVT or EVT for patients with pre-existing dementia or cognitive impairment.^76^

#### Acute ICH treatment

Recent randomized-controlled trials (RCTs) showed positive effects in certain acute surgical and medical management for ICH (e.g. intensive care bundle with blood pressure reduction, early minimal invasive hematoma evacuation, and the use of andexanet alfa in factor Xa inhibitors associated ICH), resulting in better functional outcome, reduced mortality, and reduced hematoma volume.^77^ Since poststroke cognitive impairment correlates with size of hematoma, it is likely that these treatments will result in less cognitive impairment post-ICH. To date, cognition was not assessed in those RCTs, and cognitive endpoints should be added to further trials of acute ICH treatments.

### Long-term preventive treatments

Given the strong association between vascular risk factors and cognitive impairment,^52^ measures that can improve control of vascular risk factors should prevent cognitive impairment that is associated with stroke, SVD, and/or AD. Here, we provide an update on the effects of several established pharmacological treatments (anti-hypertensive agents, lipid-lowering agents, anti-diabetic agents, anti-thrombotic agents) in the primary/secondary prevention of cardiovascular disease/stroke upon incident dementia or cognitive decline with reference to recent RCTs or meta-analysis of RCTs among at-risk subjects without dementia (if such data are available). Moreover, since vascular-related (e.g. physical activity and diet) and other non-vascular-related lifestyle factors (e.g. cognitive/social activities) may affect manifestation of VCI, recent RCTs have investigated effects of multidomain lifestyle intervention upon cognitive impairment. These recent RCTs will be discussed here. The effects of individual domain lifestyle intervention upon cognition are beyond the scope of this review.

#### Anti-hypertensive agents

Meta-analysis of recent RCTs (e.g. SPRINT-MIND) showed that blood pressure lowering with anti-hypertensives was associated with approximately 10% reduction in incident dementia/cognitive decline and the effect was greater with trials achieving a greater systolic blood pressure difference between randomized arms (e.g. ⩾ 10 mmHg).^78–82^ The SPRINT-MIND in patients with hypertension but without stroke reported that reducing blood pressure to 120 mmHg systolic was associated with a reduction in both incident MCI and a combined MCI plus dementia endpoint; it was also associated with reduced WMH progression on MRI suggesting the benefit is at least partially mediated by a reduction in SVD progression.^81^ The PROGRESS study showed that blood pressure lowering was associated with a reduced risk of incident dementia and cognitive decline.^83^ Pooled analysis including PROGRESS, ProFESS, SCOPE, ACCORD MIND, PreDIVA, and SPRINT-MIND confirmed that anti-hypertensive agents were associated with reduced WMH progression.^84^

With respect to the class of anti-hypertensive agents, a recent meta-analysis involving 649,790 participants found that the use of angiotensin II receptor blockers (ARBs) or calcium channel blockers (CCBs) was associated with reduced risk of dementia.^85^ In another study among those with MCI and hypertension, 1-year treatment with candesartan (ARB) had superior cognitive outcomes compared with lisinopril (angiotensin-converting enzyme inhibitor) and such effects were achieved independent of the blood pressure-lowering effect of candesartan.^86^ In a recent study in the IJS, Henley and colleagues investigated the impact of modulating the renin–angiotensin system upon cerebrovascular reactivity in individuals with MCI based on findings from two RCTs (n = 102) that compared candesartan with lisinopril or placebo.^87^ They found that candesartan improved cerebrovascular reactivity and these findings were independent of its blood pressure effect. In contrast, another RCT showed that treatment with losartan (ARB), amlodipine (CCB), or atenolol did not differ in their effects on cerebrovascular reactivity in sporadic SVD.^88^A meta-analysis of RCTs (n = 12,849) comparing BBB-crossing renin–angiotensin drugs with non-crossing drugs found that older adults taking BBB-crossing drugs (e.g. candesartan and lisinopril) exhibited better memory recall over up to 3 years of follow-up, relative to those taking BBB non-penetrant medications (e.g. losartan and enalapril).^89^ Taken together, these data suggest CCB and ARB are suitable agents to use in stroke patients to help reduce dementia risk, but further studies are required to determine which anti-hypertensive regimens are most effective.

#### Anti-diabetic agents

Although earlier studies showed no effects of intensive glucose-lowering upon risk of dementia/cognitive decline in diabetic patients and hypoglycemic episodes during treatment were associated with increased risk of dementia,^90,91^ meta-analysis of recent RCTs suggested that anti-diabetic agents were associated with less cognitive decline in diabetic patients.^92^ Observational studies suggested that biguanide (metformin), thiazolidinedione (e.g. rosiglitazone and pioglitazone), glucagon-like peptide-1 receptor agonists (GLP-1RA) (e.g. liragludie, exenatide, and dulaglutide), and dipeptidyl peptidase-4 (DPP-4) (e.g. sitagliptin and linagliptin) were associated with less dementia.^93–96^ The CARMELINA study, however, showed that linagliptin (DPP-4) did not modulate cognitive decline more than 2.5 years,^97^ while the REWIND study showed that the use of dulaglutide (GLP-1RA) was associated with a 14% reduction in the risk of cognitive decline over a median follow-up of 5.4 years.^98^ Smaller clinical trials using liraglutide (GLP-1RA) also yielded positive effects on cognitive outcomes among diabetic patients.^99,100^ In this issue of IJS, Adamou and colleagues conducted a systematic review and meta-analysis of cardiovascular outcome RCTs comparing GLP-1RA versus placebo in patients with or without diabetes. Among a total of 82,140 subjects of 11 RCTs, they found that GLP1-RAs demonstrated a 16% relative reduction in stroke compared with placebo.^101^ Such finding supports that GLP-1RA prevents CVD even in those without diabetes, which may explain at least partially the potential cognitive benefits of GLP-1RA. In another post hoc analysis of two RCTs among diabetic patients, semaglutide (GLP-1RA) associated with reduced incidence of any first stroke, primarily driven by prevention of small-vessel occlusion.^102^ Findings of a phase II RCT presented at the Alzheimer’s Association International Conference 2024 (July 28 to August 1) showed that the use of liraglutide (GLP-1RA) for 1 year was able to slow both brain atrophy and cognitive decline when compared to placebo among patients with early AD. Overall, both preclinical and clinical studies suggested that GLP1-1RA may exhibit beneficial impact upon the brain and cognition beyond its glucose-lowering effects.^103,104^ To date, there are limited data based on RCTs evaluating the effects of anti-diabetic agents upon SVD progression. The ACCORD MIND showed that a combination of intensive treatment of diabetes, blood pressure, and lipids among diabetic patients was associated increased WMH volume at 40 months and reduced brain atrophy. However, such differences were lost at 80 months.^105,106^ There was no difference in cognitive performance between both treatment arms at any time point. Future study is needed to confirm the effects of GLP-1RA upon the brain and cognition in at-risk individuals (e.g. early SVD/AD, mixed SVD/AD, and poststroke) among those with or without diabetes, although results to date suggest this is a very promising area.

#### Lipid-lowering agents

Despite early concerns regarding the use of statins and risk of cognitive impairment,^107^ meta-analysis of observational studies has shown that the long-term use of statins is associated with a reduced risk of dementia.^108^ A recent observational study among patients with heart failure showed that the use of statins was associated with a lower risk of all-cause dementia and its subtypes (AD, vascular dementia, and unspecified dementia).^109^ However, the majority of RCTs evaluating cognitive outcomes after statin therapy failed to show a cognitive benefit with exception of one study.^110^ Similar concerns were also raised with respect to the association between the use of proprotein convertase subtilisin–kexin type 9 (PCSK9) inhibitors and cognitive impairment.^111^ Subsequent large-scale RCTs showed that evolocumab (PCSK9) when used with statins was not associated with cognitive decline.^112,113^ A recent meta-analysis of RCTs (n = 128,691) involving contemporary lipid-lowering agents (statins, PCSK9 inhibitors, and ezetimibe) found that these agents were not associated with cognitive impairment, and that a low low-density lipoprotein cholesterol level did not influence the incidence of cognitive disorder or global cognitive performance.^114^ In another meta-analysis involving both observational studies and an RCT among stroke patients, poststroke statin use was associated with decreased risk of cognitive impairment, with a larger potential effect of higher dose and longer duration of statin use on prevention of dementia or cognitive impairment poststroke.^115^ Therefore, overall, compelling evidence has shown that lipid-lowering agents do not harm cognition.^116^ Meta-analysis including telmisartan/rosuvastatin study, ROCAS, and PROSPER showed that statin use was associated with less WMH progression and incident lacunes.^117^ Whether lipid-lowering agents may prevent incident dementia or cognitive decline in certain at-risk population (e.g. poststroke, early SVD or AD, LAD, and heart failure) requires further investigation.

#### Anti-thrombotics

Aspirin is an established treatment for secondary prevention of cardiovascular disease or non-cardioembolic ischemic stroke. It has an anti-inflammatory effect that may potentially benefit SVD or AD given both of these diseases are associated with neuroinflammation. Its efficacy and safety as a primary preventive agent for those without cardiovascular disease or stroke were investigated by the ASPREE trial, which showed that the use of low-dose aspirin (100 mg daily) over a median period of 4.7 years was not associated with reduced incident cardiovascular disease nor dementia among elderly (>70 years) who did not have cardiovascular disease/stroke, dementia, or disability at baseline. Moreover, it was associated with higher risk of major hemorrhage.^118^ In a more detailed subgroup analysis of the ASPREE, aspirin was not associated with risk of overall dementia nor with probable AD, MCI, or cognitive decline.^119^ The JPAD trial also showed that low-dose aspirin was not associated with reduced dementia among diabetic patients over a period of 7 years.^120^ Similarly, the ASCEND trial showed that the use of low-dose aspirin over a period of 7.4 years was not associated with reduced dementia among diabetic patients without history of cardiovascular disease.^121^ A recent meta-analysis on five RCTs (n = 46,804) showed that aspirin was not associated with reduced dementia, MCI, or cognitive decline.^122^ Therefore despite its established benefit in secondary prevention in cardiovascular disease and non-cardioembolic ischemic stroke, data based on RCTs have failed to show any cognitive benefit of aspirin in those without cardiovascular diseases or stroke.

Cilostazol is a phosphodiesterase (PDE)-3 inhibitor with anti-platelet properties that may also have endothelial stabilizing properties. Among subjects with subclinical SVD (confluent WMH on MRI), cilostazol was not associated with reduced progression of WMH or of cognitive decline when compared to placebo.^123^ In another RCT among subjects with mildly symptomatic SVD (confluent SVD and at least one lacune on MRI) with or without history of stroke, although cilostazol was associated with reduced incident stroke, it was not associated with reduced WMH progression when compared to aspirin.^124^ In the recent LACI-2 trial with a 2 × 2 factorial design, cilostazol alone did not reduce cognitive decline.^125^ However, the isosorbide mononitrate (ISMN)-cilostazol combination was associated with reduced composite of adverse vascular, dependence, and cognitive outcomes in patients with clinical lacunar ischemic stroke.^125^ ISMN on its own was associated with less cognitive decline. ISMN is a nitric oxide (NO) donor which augments the NO-cyclic guanosine monophosphate (cGMP) PDE5-inhibitor pathway and may improve endothelial function. The phase 3 LACI-3 trial to evaluate the effectiveness of this drug combination is on-going.

RCTs completed more than a decade ago showed that long-term dual anti-platelet therapies when compared to single anti-platelet therapy (SPS3: aspirin and clopidogrel vs aspirin; PRoFESS: aspirin plus extended-release dipyridamole vs clopidogrel) were not associated with better cognitive outcomes.^126,127^ Moreover, hemorrhagic complications were higher with long-term dual anti-platelet agents. In contrast, among AF patients, the use of oral anti-coagulants was found not only to be associated with lower risk of stroke but also with dementia,^128,129^ possibly through the prevention of subclinical infarcts or microinfarcts. A recent meta-analysis of observational studies showed that direct oral anti-coagulants (DOAC) were associated with lower risk of dementia (hazard ratio = 0.88) compared with warfarin, particularly in Asian patients and among patients younger than 75 years.^130^ RCTs are needed to confirm the superior effects of DOAC over warfarin upon reducing incident dementia/cognitive decline and to compare the efficacy of various DOACs.

#### Polypill (anti-hypertensive agents and statins)

In TIPS-3 trial in which older subjects (>65 years) with vascular risk factors were recruited, a polypill (anti-hypertensive agents and statins) with or without aspirin was not associated with reduced cognitive outcomes as measured by neuropsychological tests, but was associated with reduced functional decline over a mean follow-up period of 5 years.^131^ The reduced functional decline might possibly be partially accounted for by less cognitive decline in the polypill group, which was not be captured by the neuropsychological tests used in the study. Note further that the blood pressure was lowered by only a mean of 5.7 mmHg during the study period, which might not be sufficient to induce a benefit upon cognition.

#### Multidomain lifestyle intervention

The FINGER study showed that a multidomain lifestyle (vascular risk monitoring, Mediterranean-like diet, physical exercise, cognitive training) intervention (Table 1) could improve or maintain cognitive functioning in at-risk elderly people without dementia.^132^ Meta-analysis suggested that multidomain (e.g. physical exercise plus cognitive training) was associated with more improvement in cognitive function when compared to single-domain intervention.^133,134^ The SMARRT study recruited elderly subjects with two or more of the eight AD-modifiable risk factors that were not well controlled and randomized them into a personalized multidomain risk-reduction intervention or to general education. The study showed that after 2 years, the personalized multidomain risk-reduction intervention led to modest improvements in cognition, dementia risk factors, and quality of life.^24^ A few other similar studies also showed positive effects of multidomain intervention upon cognition.^16,19^ However, the AgeWell.de study and J-MINT study failed to show that a multidomain intervention was effective in improving cognitive performance among at-risk elderly individuals more than 2 years and 18 months, respectively.^20,23^ Subgroup analyses from the J-MINT study suggested that interventions appeared to be particularly effective for individuals with high attendance during exercise sessions, and those with APOE ε4 polymorphism and elevated plasma glial fibrillary acidic protein (GFAP) levels. The J-MIND-Diabetes study also failed to show that a multidomain intervention could prevent cognitive decline among diabetic patients with MCI.^135^ A meta-analysis including RCTs of longer duration showed that multidomain intervention could induce only slight improvement in cognitive function but was not able to reduce incident dementia.^136^ Another meta-analysis including only two RCTs among stroke patients showed that multidomain intervention improved processing speed and attention.^137^ To date, available evidence based on subgroup analysis suggested that subjects with following features may benefit more from multidomain interventions:^138^ low education,^139^ poor control in modifiable risk factors,^132,140^ subtle or subjective cognitive decline,^132,141^ minimal brain atrophy,^142^ ApoE ε4 carriers,^23,143,144^ positive AD biomarkers (e.g. Aβ, high GFAP),^23,141^ and good adherence to the lifestyle intervention.^23^ A more intensive regime may also be more effective.^20^ Future study is needed to investigate the profile of at-risk elderly individuals who may benefit most from multidomain intervention. Moreover, a reason explaining the negative studies is that the evolution from subclinical to clinical stages of SVD/AD commonly spans over years to decade(s). Therefore, it is likely that a large study of long duration is required to show a significant effect of intervention upon incident dementia. Epidemiological studies that used similar methods over sequential periods showed that dementia incidence has indeed decreased at the population level in several Western countries,^145^ which could be at least partially explained by better control of vascular risk factors, reduced burden of both cerebral SVD Aβ, and better education when compared to that of previous cohorts.^145^

**Table 1.** Recent multidomain intervention RTCs on cognitive decline.

Trial name/ID number	Region	Population/sample size	Intervention	Intervention given outcomes
TIGER, NCT03528005^15^	Taiwan, China	Community-dwelling outpatients aged 65 years or older with at least three chronic medical conditions/398 total (199 per group)	Integrated multidomain intervention (16 2-h sessions per year of exercise, cognitive training, nutrition, and disease education, plus individualized treatment) vs usual care for 12 months	Intervention group had significantly higher mean SF-36 physical component scores overall (difference = 0.8; 95% CI = 0.2–1.5; p = 0.010). SF-36 mental component scores were significantly higher in intervention group at 12 months (57.2 vs 55.3; p = 0.019)
SURPERBRAIN, NCT03980392^16^	Korea	Adults aged 60–79 years without dementia and with one or more modifiable dementia risk factors/152 total (51 FMI, 51 HMI, and 50 control)	Facility-based multidomain intervention (FMI) vs home-based multidomain intervention (HMI) vs general health advice control for 6 months	Retention rates were 88.2% (FMI) and 96.1% (HMI). Adherence was 94.5% (FMI) and 96.8% (HMI). RBANS total scale index score improved significantly in FMI (5.46 ± 7.50, p = 0.004) and HMI (5.50 ± 8.14, p = 0.004) compared with control (−0.74 ± 11.51)
Look AHEAD-MIND, NCT00017953^17^	The United States	Individuals with type 2 diabetes and overweight or obesity/3938 total (1984 intervention)	Intensive lifestyle intervention vs diabetes support and education for 10 years	No overall differences in cognitive decline rates between groups. Subgroup with baseline cardiovascular disease in the intervention arm performed worse on Stroop test
MEDEX, NCT02665481^18^	The United States	Older adults aged 65–84 with subjective cognitive concerns/585 total	Four groups: Mindfulness-based stress reduction (MBSR n = 150), exercise (n = 138), combined MBSR and exercise (n = 144), or health education control (n = 153) for 18 months	No significant effect of MBSR or exercise on episodic memory or executive function at 6 or 18 months. No significant effects at 18 months. No significant improvements in secondary outcomes (hippocampal volume, dorsolateral prefrontal cortex measures, functional cognitive capacity, self-reported cognitive concerns)
COMBAT, ChiCTR190002548.^19^	China	209 community-dwelling older adults aged 60 years or older, with ⩾2 risk factors of cognitive decline/209 total (99 intervention), 192 completed	Multidomain intervention (meditation, cognitive training, exercise, nutrition counseling) vs usual care for 9 months, with 1-year follow-up	Intervention group showed significant enhancement in cognitive performance immediately after intervention (between-group difference in Z-score = 0.20, 95% CI = 0.053–0.35; Hedges’ g = 0.40, 95% CI = 0.29–0.50). Benefit not significant at 1-year follow-up
AgeWell.de, DRKS00013555^20^	Germany	60 to 77 years and CAIDE risk score 14 of ⩾9 points/1030 participants at baseline (487 intervention), 819 completed	Multidomain intervention (nutrition, medication optimization, physical, social, and cognitive activity) vs general health advice for 2 years	No significant differences (average marginal effect = 0.010, 15.7% difference, p = 0.874) in global cognitive performance between groups. Intervention improved health-related quality of life and reduced depressive symptoms in women.
THISCE, NCT03056768^21^	Taiwan, China	Community-dwelling older adults/1054 community-dwelling older adults (529 intervention)	Multidomain intervention (exercise, cognitive training, nutritional counseling, chronic condition management) vs control for 1 year	Intervention significantly prevented cognitive declines and physical frailty, especially in those with ⩾3 impaired intrinsic capacity subdomains (MoCA: coefficient = 1.909, 95% CI = 0.736–3.083; CHS frailty scores: coefficient = −0.405, 95% CI = −0.715 to −0.095). Significant improvements in MoCA scores for those with poorer baseline cognition and vitality
GOIZ-ZAINDU, NCT06163716^22^	Spain	CU older adults, CAIDE risk score ⩾6/125 participants (61 intervention)	Multidomain intervention (MD-Int, including cardiovascular risk control, nutrition, physical activity, cognitive training) vs regular health advice (RHA) for 1 year	Higher proportion of participants declined in NTB executive function (64% RHA vs 40% MD-Int) and processing speed scores (61% RHA vs 39% MD-Int)
J-MINT, UMIN000038671^23^	Japan	Adults aged 65–85 years subjects with MCI/531 participants (265 in intervention), 406 completed the trial	Multidomain intervention (vascular risk management, exercise, nutrition, cognitive training) vs control for 18 months	No significant difference in preventing cognitive decline (between-group difference in composite score changes = 0.047, 95% CI = −0.029 to 0.124). Positive impacts on secondary health outcomes. More effective for those with high attendance in exercise sessions and those with APOE ε4 allele and elevated plasma GFAP levels
SMARRT, NCT03683394^24^	The United States	Adults aged 70–89 years with ⩾2 of 8 targeted dementia risk factors/172 total (68/82 in intervention and 81/90 in control completed)	Personalized risk-reduction goals with health coaching and nurse visits vs health education control for 2 years	Intervention group showed larger improvements in composite cognitive score (ATE of SD = 0.14; 95% CI = 0.03–0.25; p = 0.02), better composite risk factor score (ATE of SD = 0.11; 95% CI = 0.01–0.20; p = 0.03), and improved quality of life (ATE = 0.81 points; 95% CI = −0.21 to 1.84; p = 0.12)

SF-36: 36-Item Short-Form Health Survey; CI: confidence interval; FMI: facility-based multidomain intervention; HMI: home-based multidomain intervention; RBANS: Repeatable Battery for the Assessment of Neuropsychological Status; SPPB: Short Physical Performance Battery; HR: hazard ratio; MBSR: mindfulness-based stress reduction; CAIDE: cardiovascular risk factors, aging and dementia; MoCA: Montreal Cognitive Assessment; CHS: Cardiovascular Health Study; NTB: neuropsychological test battery; MD-Int: multidomain intervention; RHA: regular health advice; MCI: mild cognitive impairment; ATE: average treatment effect; SD: standard deviation; CU: cognitively unimpaired.

#### Others

Minocycline is a tetracycline antibiotic with anti-neuroinflammatory and BBB-stabilizing properties based on preclinical studies.^146–148^ However, a phase II RCT showed that minocycline was not effective in reducing neuroinflammation or BBB permeability in patients with SVD as measured by ^11^ C-PK11195 PET and dynamic contrast-enhanced MRI, respectively.^149^ DL-3-n-butylphthalide (NBP) is a synthetic compound with multiple pathological pathways related to ischemic stroke (e.g. oxidative stress, neuroplasticity, neuronal apoptosis, and autophagy). Its use at the acute stage of ischemic stroke in conjunction with reperfusion therapy was associated with improved outcomes.^150^ It was approved by China Food and Drug Administration for use in stroke. An RCT more than 24 months showed that the use of NBP was associated with improved cognitive and daily function in patients with MCI associated with SVD.^151^ A meta-analysis of 26 RCTs showed that it improved cognitive outcomes in poststroke cognitive impairment.^152^ Future studies should investigate its effects upon slowing down progression of SVD, AD, and cognitive decline in the long term.

### Limitations of current data

Much of the data discussed above is from studies examining the effect of acute stroke interventions and longer-term prevention measures to prevent the onset of cognitive decline and dementia. There are much less trial data available in patients with established poststroke dementia.^153^ Even when looking at prevention trials, much of the data is from small underpowered studies with risk of bias, and larger, well-powered RCTs looking at prevention of dementia in patients with stroke are needed.^153^ Studies such as SPRINT-MIND have demonstrated the benefit of adding cognitive outcomes to large RCTs with other cardiovascular endpoints, and this approach needs to be adopted widely in stroke trials.

### Symptomatic treatment in poststroke dementia and VCI

The treatments discussed above may reduce cognitive impairment by preventing brain damage, but are their effect treatment to improve cognition in those with established VCI and vascular dementia? Both cholinesterase inhibitors and memantine are used as symptomatic treatments in AD, and it has been suggested they may have benefit in VCI perhaps due to denervation resulting in loss of cholinergic innervation. Their benefit was rigorously examined in the recent European Stroke Organisation Guidelines which concluded that “there is no robust data that pharmacological interventions including cholinesterase inhibitors and memantine improved symptoms or delayed progression to dementia.”^153^ Almost all data were from trials of dementia rather than poststroke cognitive impairment and interpretation of these trials is complex, in particular because many cases labeled as vascular dementia may in fact have mixed dementia which is the predominant dementia type in the elderly. Indeed, there has been debate as to whether any benefit reported with cholinesterase inhibitors in vascular dementia trials is due to a true effect on vascular dementia, or an effect on concurrent Alzheimer’s pathology. To address this question, the efficacy of donepezil was examined in a RCT in a model of pure vascular dementia, CADASIL. Although there was a significant effect on the secondary endpoint of executive dysfunction, there was no improvement in the primary cognitive endpoint or in activities of daily living.^154^ Therefore, currently, there is no clinical indication for cholinesterase inhibitors and memantine in poststroke cognitive impairment or vascular dementia, although they can be used in cases of suspected mixed dementia with co-existent AD.

Some studies have examined non-pharmacological interventions including acupuncture, transcranial magnetic stimulation, and cognitive interventions but these have all been small and have not proven benefit of any specific interventions.^155^

### Management of cognitive symptoms associated with dementia

VCI is often associated with other symptoms including depression, apathy, and emotionalism. It is important to look for depression which may respond well to treatment with anti-depressants and cognitive interventions. While apathy may co-exist with depression, the two are quite distinct, and apathy may occur without depression. Apathy is a reduction in goal-directed activity in the cognitive, behavioral, emotional, or social domains of a patient’s life and occurs in one of three patients after stroke.^45^ It can be differentiated from depression by its manifestation as a reduction in initiative, while depression manifests as negative emotionality. It is particularly common in SVD, and recent data suggest it results from damage to brain networks from white matter tract disruption, by a similar mechanism to that which can cause VCI.^156^ No drug treatments have been proven to improve apathy after stroke, and a recent secondary analysis of EFFECTS trial data showed no benefit of fluoxetine in preventing development of apathy after stroke.^157^ Current management is limited to psychological support to help the patient, and particularly the carer, adapt to this disabling symptom.

A related symptom is poststroke emotionalism which is also common, occurring in one in five stroke patients and in those with VCI, particularly due to SVD. This topic is covered by an excellent review by Broomfield and colleagues in this issue of IJS. They describe how TEARS-Q (Testing for Emotionalism after Recent Stroke–Questionnaire) has recently been developed to allow standardized assessment. Treatment options are limited, and there have been few adequately powered treatment trials. Anti-depressants may reduce severity, but more trial data are required. There have been no RCTs of non-pharmacological interventions.^158^

## Conclusion

Worldwide, around 50 million people live with dementia, and this number is projected to triple by 2050, rising particularly in LMIC where around two-thirds of people with dementia live.^3^ Vascular factors contribute to many of these cases. It has been estimated 20% of all dementia cases have a predominant vascular pathology—so 10 million worldwide—while perhaps another 20% of vascular diseases may contribute to a mixed dementia picture. Therefore, the vascular contribution to dementia affects 20 million people currently and will increase markedly in the next few decades particularly in LMICs. Despite this enormous health and economic impact, it has been a neglected research area, with limited adequately powered trials of therapies, resulting in few proven treatments. Current management of VCI emphasizes prevention and treatment of stroke and vascular risk factors, with intensive hypertension control being the most robust treatment in preventing cognitive and radiological decline due to SVD and possibly preventing development of AD. Early IVT/EVT in acute stroke may also attenuate the risk of VCI. Novel treatment strategies that target cellular and molecular pathophysiology of cSVD are actively being evaluated and will hopefully lead to new disease course-modifying therapies. Meanwhile, we need an increased research focus and funding for this important area.
